# Evaluation of the Relationship Between Nitrate Use and the Prevalence of Colorectal Cancers in the United States

**DOI:** 10.7759/cureus.84530

**Published:** 2025-05-21

**Authors:** Alex N Egbuchiem, Okelue E Okobi, Oluwadamilola D Odutola, Christiana A Igbenabor, Uzoma N Okey-Ndeche, Oluwatobiloba Omotunde, Tosin Ayantoyinbo, Obinna C Abonyi, Oghenemaro O Oghotuoma, Ogechukwu H Nnabude, Chuka G Nwume

**Affiliations:** 1 Public Health Research, College of Public Health, University of Nebraska Medical Center (UNMC), Omaha, USA; 2 Family Medicine, IMG Research Academy and Consulting LLC, Homestead, USA; 3 Family Medicine, Palm Springs Campus, Larkin Community Hospital, Miami, USA; 4 Family Medicine, Lakeside Medical Center, Belle Glade, USA; 5 Internal Medicine, Medical University of South Carolina, Florence, USA; 6 Internal Medicine, St. John’s Episcopal Hospital, New York, USA; 7 Internal Medicine, Kingston Public Hospital, Kingston, JAM; 8 Internal Medicine, American International School of Medicine, Atlanta, USA; 9 Internal Medicine, BronxCare Health System, New York, USA; 10 Internal Medicine, Obafemi Awolowo College of Health Sciences, Olabisi Onabanjo University, Ago-Iwoye, NGA; 11 Internal Medicine, Spartan Health Sciences University, St. Lucia, UMI; 12 General Internal Medicine, Walsall Healthcare NHS Trust, Walsall, GBR; 13 Dermatology, Psychiatry, Cardiology, Internal Medicine, Family Medicine, Windsor University School of Medicine, Houston, USA; 14 Internal Medicine, St. John Episcopal Hospital, New York, USA

**Keywords:** alkyl nitrites, colorectal cancer, colorectal cancer, colorectal cancer (crc), correlation, ecological study, nitrate exposure, nitrates, public health and safety, united states

## Abstract

Background

Exposure to nitrate through contaminated drinking water has been suggested as a potential risk factor for colorectal cancer (CRC). However, ecological evidence across the U.S. states remains limited. This study aims to examine the association between average nitrate concentrations and CRC incidence across 31 U.S. states.

Methods

An ecological analysis was conducted using data from the Centers for Disease Control and Prevention’s (CDC's) Environmental Public Health Tracking Network and United States Cancer Statistics (2017-2021). Simple linear regression and Pearson correlation analyses were performed, stratified by gender.

Results

No significant associations were observed between nitrate concentrations and CRC incidence in both strata. Correlations were weak and non-significant.

Conclusions

In our study, state-level nitrate exposure was not significantly linked to CRC incidence. Further individual-level studies are recommended.

## Introduction

Nitrate compounds are widely used as chemical fertilizers to increase crop yields [1]. While nitrates have been extensively used, their intensive use has led to serious environmental and public health concerns, mainly because they tend to contaminate surface water and groundwater sources [2]. Increasing nitrate levels in drinking water have become a perennial problem in many rural and agricultural regions throughout the United States [3]. The contamination harms water quality, and if sustained over long periods of time, it presents health concerns from nitrate exposure.

When ingested, nitrates (NO₃⁻) from drinking water or food are initially reduced to nitrites (NO₂⁻) by bacteria in the oral cavity and gastrointestinal tract. These nitrites can react with amines and amides present in the digestive system to form N-nitroso compounds (NOCs), a class of chemicals that includes many known carcinogens. The formation of NOCs is particularly favored in acidic environments such as the stomach. Some NOCs can damage DNA and promote mutations, processes which may contribute to the development of colorectal cancer (CRC). Diets high in red and processed meats, which are rich in amines and amides, can further enhance NOC formation when combined with elevated nitrate or nitrite intake. While this molecular pathway is biologically plausible and supported by laboratory evidence, epidemiological studies have yielded mixed results regarding the direct association between nitrate exposure from drinking water and CRC risk [4].

One of the major cancer burdens in the United States is CRC. The American Cancer Society [5] notes that CRC is the third most commonly diagnosed cancer and the second most common cause of cancer death for both men and women. Despite the progress that has been made in screening, early detection, and treatment, the prevalence of CRC remains a concern, particularly in certain geographical regions and populations [6]. The pathogenesis of CRC varies by the anatomical location of the tumor, with distinct molecular characteristics and histological features observed between right-sided and left-sided colon cancers [7]. These differences in tumor origin lead to variations in gene expression and mutation profiles, which in turn influence prognosis. Right-sided tumors are more frequently associated with BRAF mutations and microsatellite instability (MSI), and are often seen in individuals with a genetic predisposition to CRC. In contrast, left-sided tumors typically exhibit chromosomal instability (CIN) and a gene expression profile marked by activation of the epidermal growth factor receptor (EGFR) pathway [8,9,10]. For effective public health interventions, however, it is important to understand the environmental and lifestyle factors that contribute to the risk of CRC.

Recent studies have begun to look into the relationship between nitrate exposure and the development of CRCs. Though nitrates themselves are relatively inert, on ingestion, the body can convert nitrates to nitrites (also carcinogenic) and finally to NOCs, many of which are known carcinogens. Several epidemiological studies have indicated that chronic consumption of nitrate‑contaminated water or nitrate‑rich foods, especially when combined with diets high in red and processed meats, may contribute to CRC. For example, studies [11,12] reported an increased risk of CRC associated with nitrate levels in drinking water.

Large-scale, population-level studies that examine these ecological relationships between nitrate contamination and CRC prevalence in the United States have a notable gap. Although laboratory studies and some cohort analyses have yielded important insights, comprehensive data linking patterns of nitrate use, environmental contamination, and CRC prevalence at regional or national scales are currently lacking [11-12]. Furthermore, most existing studies emphasize dietary nitrate from processed foods rather than exposures through drinking water, possibly exacerbated by rice with agricultural runoff.

Recent findings indicate that not all forms of CRC respond the same way to nitrate exposure. Variations among CRC subtypes, such as differences in genetic mutations or tumor biology, could influence how these cancers develop or progress in response to dietary nitrates. This underscores the importance of considering cancer subtypes when evaluating environmental risk factors.

This study aims to evaluate the association between nitrate exposure measured through water contamination and fertilizer use and the prevalence of CRC across various regions in the United States. Understanding this connection is critical for informing regulations on fertilizer use, improving water quality standards, and providing guidance on the public health strategies for reducing the burden from CRC.

## Materials and methods

Study design

This study employed an ecological design to evaluate the association between nitrate exposure through community drinking water systems and the prevalence of CRC across U.S. states. The analysis was based on publicly available aggregated data for the years 2017-2021.

Data sources

Nitrate exposure data were obtained from the Centers for Disease Control and Prevention (CDC) Environmental Public Health Tracking Network, specifically the dataset on Nitrate Concentrations in Community Water Systems [13]. The original dataset covered the period from 2006 to 2023. To ensure temporal consistency with the CRC prevalence data, nitrate measurements were filtered to retain only records from 2017 to 2021. For each state, the nitrate concentrations from these five years were aggregated by calculating the mean, resulting in a single average nitrate concentration value per state.

CRC prevalence data were retrieved from the United States Cancer Statistics (USCS) database managed by the CDC [14]. The dataset provided age-adjusted prevalence rates for CRC at the state level, aggregated for the years 2017 to 2021.

Due to limitations in data availability, the analysis was restricted to 31 states for which both complete CRC prevalence and nitrate exposure data were available. These states are Arizona, California, Colorado, Connecticut, Delaware, Florida, Iowa, Kansas, Kentucky, Louisiana, Maine, Maryland, Massachusetts, Michigan, Minnesota, Missouri, Nebraska, New Hampshire, New Jersey, New Mexico, New York, North Carolina, Oregon, Pennsylvania, Rhode Island, South Carolina, Tennessee, Utah, Vermont, Washington, and Wisconsin. These states together provide a broad geographic representation of the United States for this study.

Exposure assessment

For each state, the mean nitrate concentration (measured in milligrams per liter, mg/L) across all reported community water systems was calculated for the years 2017-2021. States with incomplete nitrate reporting for 2017-2021 were excluded from the analysis.

Outcome assessment

The primary outcome was the age-adjusted incidence rate (AAIR) of CRC per 100,000 persons in each state for the years 2017-2021. AAIR was used as the outcome to allow fair comparisons of CRC risk across states, controlling for differences in age distribution. This adjustment ensures that differences in cancer rates are not simply due to variations in the age structure of state populations.

Statistical analysis

Descriptive statistics were used to summarize the distribution of average nitrate concentrations (mg/L) and AAIR for CRC across the included states. Means, standard deviations, minimum, and maximum values were calculated for both variables. To evaluate the association between nitrate exposure and CRC prevalence, a simple linear regression analysis was performed. In the model, the state-level average nitrate concentration served as the independent variable, while the AAIR per 100,000 population was the dependent variable. Additionally, a Pearson correlation coefficient (r) was calculated to assess the strength and direction of the linear relationship between average nitrate levels and CRC prevalence rates. Statistical significance was set at the 5% level. All statistical analyses were performed using Stata version 18.0.

## Results

Descriptive statistics

Descriptive Statistics for Males

Table 1 provides the descriptive statistics for the AAIR of CRC per 100,000 population and nitrate concentrations among males across 31 states.

**Table 1:** Descriptive statistics for males. Obs., number of observations (31 states); Mean, the average value across observations; Std. Dev., standard deviation, indicating how much the data varies from the mean; Min., the smallest observed value; Max., the largest observed value

Variable	Obs.	Mean	Std. Dev.	Min.	Max.	*P*-value
Age-adjusted incidence rate (AAIR) of CRC per 100,000 pop.	31	40.197	4.398	32.5	53.1	<0.05
Nitrate (mg/L)	31	1.177	0.682	0.269	3.151	<0.05

According to the findings, the mean AAIR of CRC was 40.2 cases per 100,000 population, with a standard deviation of 4.4, ranging from 32.5 to 53.1 cases per 100,000. The average nitrate concentration in community water systems was 1.18 mg/L, with a standard deviation of 0.68 mg/L, and values ranging from 0.27 to 3.15 mg/L.

Descriptive Statistics for Females

Table 2 presents the descriptive statistics for the AAIR of CRC per 100,000 population and nitrate concentrations among females across 31 states.

**Table 2:** Descriptive statistics for females. Obs., number of observations (31 states); Mean, the average value across observations; Std. Dev., standard deviation, indicating how much the data varies from the mean; Min., the smallest observed value; Max., the largest observed value

Variable	Obs.	Mean	Std. Dev.	Min.	Max.	*P*-value
Age-adjusted incidence rate (AAIR) of CRC per 100,000 pop.	31	31.555	3.165	26.3	39.6	<0.05
Nitrate (mg/L)	31	1.177	0.682	0.269	3.151	<0.05

The mean AAIR of CRC was 31.6 per 100,000 population, with a standard deviation of 3.2, and ranged from 26.3 to 39.6 cases per 100,000. The average nitrate concentration in community water systems was 1.18 mg/L, with a standard deviation of 0.68 mg/L, and ranged from 0.27 to 3.15 mg/L. These results provide a general overview of CRC burden and nitrate exposure among females in the study population.

Correlation analysis

Correlation Analysis for Males

Table 3 presents the Pearson correlation analysis between average nitrate concentrations and CRC incidence rates among males across 31 states.

**Table 3:** Correlation analysis for males. -: intentionally left blank. Notes: Statistical significance levels are indicated by asterisks: *P* < 0.05 (*), *P* < 0.01 (**), and *P *< 0.001 (***). A single asterisk (*) indicates results significant at the 5% level (there is less than a 5% probability that the result is due to chance), two asterisks (**) indicate significance at the 1% level, and three asterisks (***) indicate significance at the 0.1% level.

Variables	AAIR	Nitrate (mg/L)
AAIR	1	-
Nitrate (mg/L)	0.122	1
N	31	-
*P*-value	<0.05	<0.05
Significance level	^*^P < 0.05, ^**^P < 0.01, ^***^P < 0.001	

The findings reported a correlation coefficient of *r *= 0.122, suggesting a very weak positive relationship between nitrate exposure and CRC incidence rates among males. This association was also not statistically significant at 5% level of significance.

Correlation Analysis for Female

Table 4 shows the Pearson correlation analysis between average nitrate concentrations and CRC incidence rates among females across 31 states.

**Table 4:** Correlation analysis for female. -: intentionally left blank. Notes: Statistical significance levels are indicated by asterisks: *P* < 0.05 (*), *P* < 0.01 (**), and *P *< 0.001 (***). A single asterisk (*) indicates results significant at the 5% level (there is less than a 5% probability that the result is due to chance), two asterisks (**) indicate significance at the 1% level, and three asterisks (***) indicate significance at the 0.1% level.

	AAIR	Nitrate (mg/L)
AAIR	1	-
Nitrate (mg/L)	0.200	1
N	31	-
*P*-value	<0.05	<0.05
Level of significance	^*^P < 0.05, ^**^P < 0.01, ^***^P < 0.001	

The correlation coefficient was *r* = 0.20, indicating a weak positive relationship between nitrate exposure and CRC incidence rates among females. However, the correlation was not statistically significant at 5% level of significance.

Simple Linear Regression Models 

Table 5 presents the results of simple linear regression models examining the association between average nitrate concentration in community water systems and CRC incidence rates, stratified by gender.

**Table 5:** Simple linear regression models examining the association between nitrate exposure and colorectal cancer incidence, stratified by gender. -: intentionally left blank. Note: Statistical significance levels were *P* < 0.05; three asterisks (***) indicate significance at the 0.1% level. R² : measures the proportion of variance in the dependent variable (age-adjusted incidence rate, AAIR) that is explained by the independent variable (average nitrate).

AAIR	Male	Female
Average nitrate (mg/L)	0.789	0.928
-	(0.848)	(0.689)
Constant	39.268^***^	30.463^***^
-	(1.365)	(1.021)
Observations	31	31
*R*^2^	0.015	0.040
Adjusted *R*^2^	-0.019	0.007
*P*-value	<0.05	<0.05

The aforementioned regression findings showed that there was a positive coefficient (β = 0.79, *P *> 0.05) for average nitrate concentration with AAIR for the males. It is evident that there exists a positive but non-significant relationship between the two at the 5% level of significance. Furthermore, its *R*² value was 0.015, indicating that nitrate exposure accounted for only 1.5% of the variability in the AAIR among males.

Similarly, the females revealed a positive relationship between average nitrate concentration with AAIR, which was not statistically significant at 5% level of significance (β = 0.93, *P *> 0.05). The model also reported an *R*² value of 0.04, meaning that only 4% of the variation in CRC incidence among females was explained by nitrate exposure. Overall, these findings indicate that in both males and females, no significant association was observed between nitrate concentrations in drinking water and AAIR at the state level.

## Discussion

In this study, we evaluated the association between nitrate exposure in community water systems and CRC prevalence rates across 31 U.S. states, stratified by gender. Overall, the results showed no statistically significant relationship between average nitrate concentrations and AAIR among both males and females.

The simple linear regression models demonstrated very low *R*² values, indicating that nitrate exposure explained little of the variation in AAIR. Additionally, Pearson correlation coefficients were weak and non-significant in both genders. These findings suggest that, within the observed nitrate concentration ranges and over the studied period (2017-2021), nitrate levels in public water systems may not be strongly associated with CRC prevalence at the state level.

However, mechanistic and molecular-level evidence suggests that nitrate and nitrite exposure, especially from dietary sources such as processed meats, can initiate and promote CRC development under certain physiological conditions. Nitrates (NO₃⁻) and nitrites (NO₂⁻), though chemically inert, can be reduced endogenously into NOCs, a class of known carcinogens. These compounds can cause DNA mutations, impair DNA repair, and promote malignant transformation, particularly in colonic tissues exposed to chronic inflammation or excess nitric oxide signaling via inducible nitric oxide synthase (iNOS) activity [15].

Nitrite-derived nitric oxide has been shown to have dual, concentration- and stage-dependent effects on CRC cells. At low levels, nitrite can inhibit early-stage tumor growth, whereas at higher levels it may enhance tumor progression in late-stage cells [16]. The molecular environment also modulates this risk - heme iron and protein-rich diets increase endogenous nitrosation, while polyphenols and antioxidants in vegetables inhibit this conversion [17].

Though our ecological findings showed no strong association between waterborne nitrate and CRC prevalence, dietary intake, particularly from processed meats, has shown more consistent associations in meta-analyses and cohort studies. Another study [18] found that individuals with high N-nitrosodimethylamine (NDMA - a potent NOC) intake had a significantly increased CRC risk. Similarly, the World Cancer Research Fund (2018) recommends limiting red and processed meat consumption due to their strong association with CRC. Finally, ecological analyses at the state level are inherently limited by potential aggregation bias and may mask individual-level associations. Although the analysis is limited to 31 states, the geographic diversity and demographic variability of the included states enhance the generalizability of the findings to the broader U.S. context. Future research should integrate molecular biomarkers, dietary sources, and individual exposure histories into longitudinal studies to clarify these associations further.

Limitations

Our study has several important limitations. First, as a retrospective ecological analysis using state-level aggregated data, the study is inherently susceptible to ecological fallacy, meaning that associations observed at the group level may not accurately reflect individual-level risks. Second, the design is based on simple correlation and regression analyses, which evaluate association but cannot establish causality. Therefore, no causal relationship between nitrate exposure and CRC prevalence can be inferred from these results. Third, the study included only 31 states, leading to a relatively small sample size and potentially reduced statistical power to detect significant relationships. Fourth, the range of nitrate exposure across the included states was narrow and predominantly below the Environmental Protection Agency’s (EPA's) maximum contaminant levels, limiting the ability to assess effects at higher exposure levels. Fifth, the study did not adjust for key confounding factors, such as dietary habits (e.g., intake of red and processed meat), socioeconomic status, smoking rates, obesity prevalence, or access to CRC screening, all of which could significantly influence cancer prevalence. Sixth, potential measurement error exists because nitrate concentrations were averaged at the state level without accounting for individual water source variations, and individuals may consume bottled water or private well water, which are not captured in the data. Seventh, there is a potential temporal mismatch because the analysis focused on recent nitrate exposure (2017-2021), whereas CRC development typically occurs over decades, meaning past exposures could be more relevant. Finally, the study did not explore possible threshold effects or non-linear dose-response relationships, which could mask associations observable only at higher exposure levels. Future research should address these limitations by utilizing individual-level, longitudinal data, incorporating broader exposure ranges, adjusting for major confounders, and considering latency periods that are appropriate for cancer development.

## Conclusions

This study assessed the association between nitrate exposure from community water systems and colorectal cancer prevalence across 31 U.S. states. The findings demonstrated no significant relationship between average nitrate concentrations and colorectal cancer prevalence rates, even when stratified by gender. Although nitrate contamination remains an important public health concern, these results suggest that, at the exposure levels observed, nitrate may not be a major contributor to colorectal cancer risk at the state level. Future research incorporating individual-level exposure data and additional risk factors is recommended to better understand potential associations.
